# Exploratory analysis of the spatial distribution of adult glioma age-adjusted county incidence rates, Nebraska Medicine, 2009–2019

**DOI:** 10.1093/nop/npad050

**Published:** 2023-08-25

**Authors:** Kendra L Ratnapradipa, Amulya Yellala, Nicole Shonka

**Affiliations:** Department of Epidemiology, University of Nebraska Medical Center, 984395 Nebraska Medical Center, Omaha, Nebraska, USA; Department of Internal Medicine, Division of Oncology and Hematology, University of Nebraska Medical Center, Omaha, Nebraska, USA; Department of Internal Medicine, Division of Oncology and Hematology, University of Nebraska Medical Center, Omaha, Nebraska, USA

**Keywords:** glioma, age-adjusted incidence rates, spatial epidemiology, cancer

## Abstract

**Background:**

Central nervous system (CNS) cancers including gliomas have low incidence but high mortality. The age-adjusted incidence rate for CNS cancers is higher in Nebraska than nationally. This exploratory study was motivated by glioma patient inquiries about possible clustering of cases within the state to see if more in-depth investigation was warranted.

**Methods:**

Using electronic health records from Nebraska Medicine, we identified Nebraska adult (age ≥19) glioma patients diagnosed between January 1, 2009 and November 1, 2019. Patient residential addresses were geocoded, mapped, and combined with annual US Census data to compute age-adjusted incidence rates (AAIR) at the county level. Counties with fewer than five cases were excluded to protect patient identity. ArcGIS software was used for geocoding and mapping.

**Results:**

Of the 285 cases included in the analysis, 53.2% were geocoded with exact match and the remainder were processed manually. Cases occurred in 47 of the 93 counties. After data suppression, 11 counties (228 cases) visually clustered in eastern and central Nebraska with AAIR ranging from 0.85 to 5.66 per 100 000.

**Conclusions:**

Many counties in the state were excluded from analysis of this rare cancer due to the small number of cases leading to unstable rates and the need to suppress data to protect patient privacy. However, this preliminary study suggests that glioma incidence is highest in central and eastern Nebraska. Next steps include analysis of state cancer registry data to ensure more complete case ascertainment.

Central nervous system (CNS) cancers in the United States have low incidence but high mortality compared to other cancers. In 2023, there will be an estimated 24 810 new diagnoses and 18 990 deaths.^1^ Gliomas are the most common adult primary intracranial tumor representing 81% of malignant brain tumors.^2^ Glioblastoma is the most common histology (about 45% of all gliomas) and has a 5-year relative survival of about 5%.^2^ The age-adjusted 2020 incidence rate for CNS cancers was higher in Nebraska than in the United States (7.0 vs. 6.4 per 100 000, respectively).^3^

Malignant glioma is incurable. Thus far, the only definite environmental exposure and risk factor for glioma is ionizing radiation, either from therapeutic radiation or secondary to atomic bomb exposure^.2^ Association between glioma risk and some specific agricultural pesticide exposures such as nitrates have been reported for male farmers, though results are controversial.^4–6^ Studies of occupational exposures have implicated agriculture workers, especially those working with herbicides, pesticides, and fertilizers as at higher risk. A cohort study of 2370 members of a Swedish Horticultural Society from 1965 to 1982 found a 3-fold and 5-fold risk of all types of brain tumors in gardeners and orchardists, respectively.^7^ The Shanghai Cancer Registry reported on 276 women who developed brain tumors from 1980 to 1984 and found an elevated standardized incidence ratio among grain farmers (standardized incidence ratio = 6.5, 95% CI = 1.3–19.1).^8^ A large Italian case–control study conducted interviews in 1984–1985 with 240 glioma cases and 465 non-glioma CNS tumors and 277 non-tumor neurologic illness patients as controls and found a 1.6 relative risk (RR) for farmers to develop glioma, specifically increased if they used chemicals to farm (RR 2.0 vs. no chemicals at RR 1.0).^9^ Khuder et al. published a meta-analysis of brain cancer risk in farmers using publications indexed on Medline from 1982 to 1997, wherein the RR of a farmer developing brain cancer was 1.3.^10^ In compilation, these works present a compelling case for an elevated risk in agricultural areas, such as the majority of Nebraska. A large portion of Nebraska sits over the High Plains Aquifer, which previous interpolation studies have identified as having high concentrations of nitrate and uranium-contaminated groundwater.^11^

Nebraska is a rural state with nearly half the population concentrated in the eastern metropolitan areas of Omaha and Lincoln. Of the 93 counties, 31 are classified as frontier (<7 residents per square mile) and another 48 are rural.^12^ Farming and ranching are major industries in the state. Of the 13 cancer centers in the state, Nebraska Medicine’s Fred and Pamela Buffett Cancer Center is the only National Cancer Institute-designated cancer center and its catchment area is the entire state. Based on anecdotal patient concerns about case clustering of gliomas, this exploratory study was conducted as a preliminary analysis to see if further investigation was warranted.

## Methods

This study was approved by the University of Nebraska Medical Center Institutional Review Board (#910-19-EP) as health records research with minimal risk which did not require patient consent.

### Data Sources

**Figure 1.**
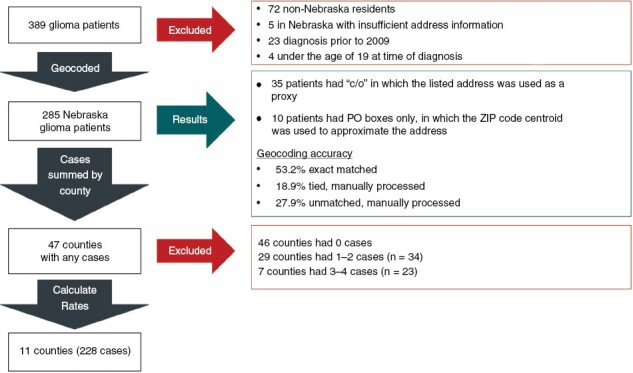
Study inclusion/exclusion flow chart.

The primary data source was the electronic health record at Nebraska Medicine. We drew a census of all identified adult (age 19 and older) glioma patients diagnosed and treated between January 1, 2009 and November 1, 2019 using ICD-10 code C71.9 (glioma). We then removed any non-glioma tumors and retained all astrocytoma, oligodendroglioma, and ependymoma of any grade. We next excluded any cases diagnosed prior to 2009 and any individuals diagnosed before the age of 19 (Figure 1). Non-Nebraska residential addresses at time of diagnosis were also excluded from analysis.

Annual US Census population estimates for each year of the study were used as denominators to calculate county-level rates. The US 2000 standard population was used to calculate age-adjusted incidence rates (AAIR), as was used in the Central Brain Tumor Registry of the United States (CBTRUS) report.^13^

### Geocoding and Data Suppression

Patient residential addresses were geocoded and mapped using ArcGIS software. Addresses with PO boxes used the ZIP Code centroid to approximate residential locations. Addresses listed as “c/o” (care of) another individual used that address as a proxy. Because analysis was conducted at the county level, individuals with incomplete addresses were imputed a county designation based on the city if present (*n* = 5). Maps were created using natural breaks (Jenks) with 5 categories.

Data were suppressed for geographic units with fewer than 5 cases due to rate instability and to protect patient privacy. These counties were excluded from rate-based analysis.

### Analysis

**Figure 2.**
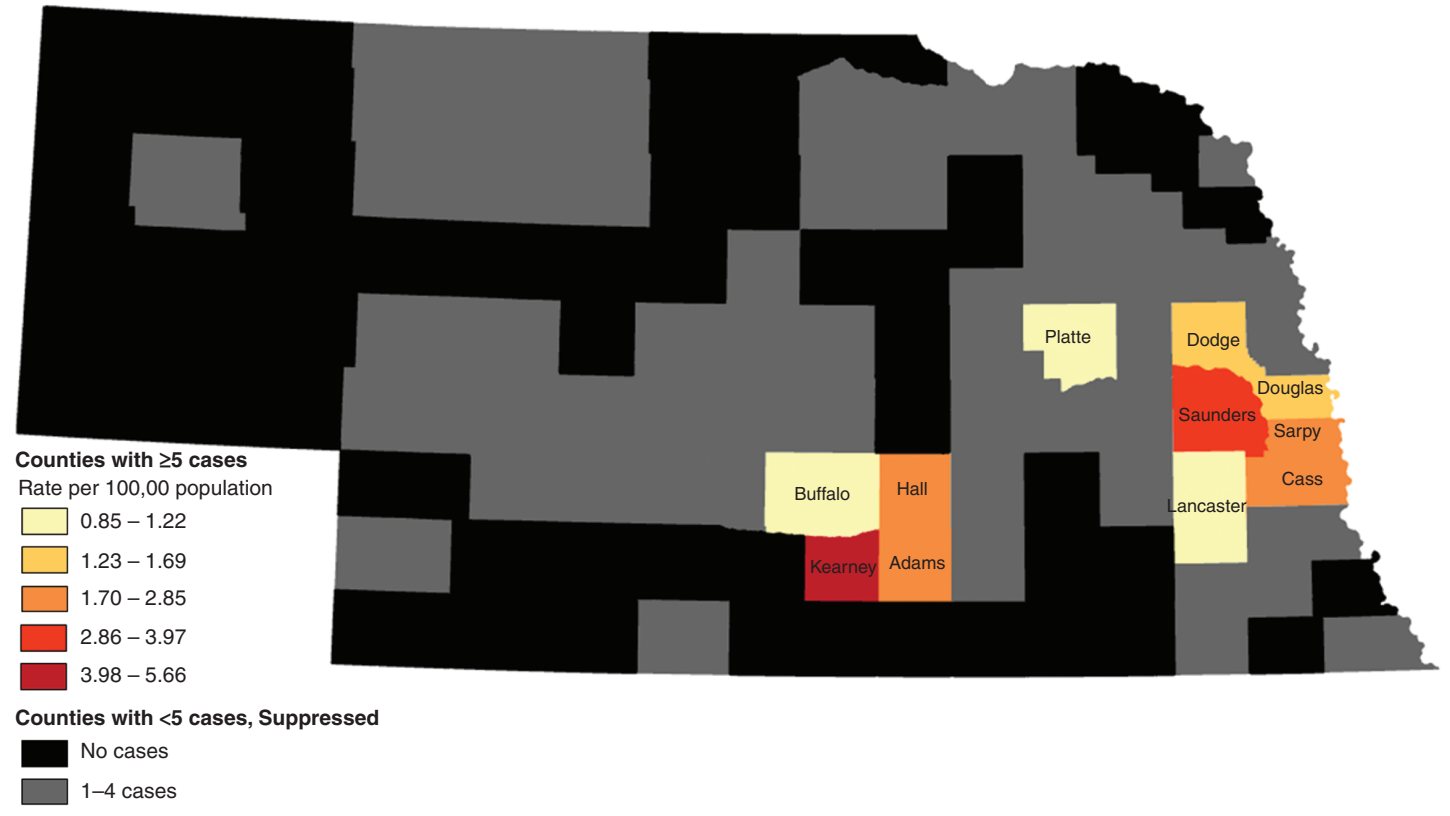
County-level adult glioma age-adjusted incidence rates, Nebraska, 2009–2019. *Data source*: Nebraska Medicine

Data were aggregated across the full 11-year study period to protect patient identity. The AAIR was calculated for each county with 5 or more cases by summing cases by 5-year age group and dividing by the age-specific summed annual county population, then multiplying by the US standard population age weights. Overall county-level AAIR was the sum of county-level age category rates. County-level maps of cumulative counts (not shown) and period AAIR (Figure 2) were used to look for spatial patterns. Sensitivity analysis (results not shown) examined AAIR by census tract with data suppression set at <2 cases.

## Results

Of the 389 glioma patients identified in the electronic medical records, 72 were non-Nebraska residents, 5 lacked sufficient address information, 23 were diagnosed prior to 2009, and 4 were under the age of 19 (age of majority in Nebraska) at time of diagnosis, resulting in 285 cases for analysis (Figure 1). Exact match for geocoding accounted for 53.2% of addresses, with the remainder manually processed.

Most cases were White (90.3%), non-Hispanic (94.8%), and male (52.9%). Patient age at diagnosis ranged from 19 to 87 years with a mean of 54.8 (standard deviation 16.6); when grouped by 5-year age categories, ages 60–64 and 65–69 were most common, each representing 13.3% of the patients. Grade 4 astrocytoma was the most common diagnosis (Table 1).

**Table 1.** Distribution of Histology and Grade for Study Sample Compared to CBTRUS

Histology	Astrocytoma	CBTRUS*	Oligodendroglioma	CBTRUS*	Ependymoma	CBTRUS*
Grade 1	0.7%	5.0 %				
Grade 2	10.7%	7.1%	3 (1.0%)	3.5%	3 (1.0%)	6.5% **
Grade 3	9.3%	6.6%	0	1.8%	2 (0.7%)	
Malignant glioma NOS	2.8%	7.8%				
Grade 4	73%	59.2%				
NOS	0.3%	0.6%				

*Central Brain Tumor Registry of the United States, 2015–2019^13^

**All ependymal tumors combined.

Cases occurred in 47 of Nebraska’s 93 counties (counts 1–97). Data were suppressed for 36 counties with <5 cases (29 counties had 1–2 cases, and 7 counties had 3–4 cases, see Figures 1 and 2), leaving 11 counties (228 cases) clustered in eastern and central Nebraska with AAIR ranging from 0.85 to 5.66 per 100 000 (Figure 2, Table 2). In the sensitivity analysis, all data at census tract level were suppressed with <5 cases. When analyzed by the 61 census tracts with 2–4 cases, an inverse “U” was apparent with outliers (not shown to protect privacy).

**Table 2.** County Age-Adjusted Adult Glioma Incidence Rates, Nebraska Medicine, 2009–2019

County by Regional Cluster	Age-Adjusted Incidence Rates per 100 000 Population
*Central*	
Adams	2.85
Kearney	5.66
Buffalo	1.14
Hall	1.96
*Eastern/Metro*	
Lancaster	0.85
Cass	2.26
Douglas	1.60
Sarpy	1.98
Saunders	3.97
Dodge	1.69
*Northeast*	
Platte	1.22

Results are only shown for counties that had 5 or more cases during the 11-year study period.

## Discussion

When limiting analysis to counties with at least 5 cases, this preliminary study suggests that glioma cases may be clustered in central and eastern Nebraska, an area characterized by the metropolitan areas of Lincoln and Omaha and farming counties around the Platte River. However, when examining the considerable number of counties with 1–4 cases, the picture is less clear, with cases spread across more of the state. Glioma is a relatively rare diagnosis and 20% of cases in the present study were excluded from county-level analysis due to data suppression of counties with fewer than 5 cases. None of the 11 county-level AAIG in this study were as high as the overall state AAIG in 2020 of 7.0 per 100 000.^3^

Previous studies of groundwater in Nebraska found concentrated agricultural pesticides and uranium contamination.^11^ Our study of the spatial distribution of incident cases is inconclusive, largely due to the small number of cases and data suppression issues. Our next step is planned cluster analysis of statewide cancer registry data to ensure statewide case ascertainment and a longer study period. A more comprehensive spatial analysis is warranted to help identify areas of potential elevated risk prior to analytical studies exploring possible etiological factors.

This study had several limitations, including being an electronic medical record review from a single institution. While the majority of glioma patients in the state are seen at this facility by the only fellowship-trained neuro-oncologist in the state, some cases may be seen at other locations including in other states, leading to incomplete case ascertainment of incident gliomas. Additionally, the study was based on address at time of diagnosis. Due to potentially long latency periods in the development of IDH mutant glioma, future etiological studies should attempt to obtain residential address histories as the residence at diagnosis may not reflect the residence during exposure window(s). Based on our findings, we plan to add residential history to data gathered for CNS tumors within our Integrated Cancer Repository for Cancer Research (iCaRe^2^) registry. Another limitation was the small number of cases for this rare cancer leading to unstable rates in less populous counties and the need to suppress data to protect patient confidentiality.

## Data Availability

This study utilized protected patient health information and the data are therefore not available for release.
